# Response to “Twitter-Based Journal Clubs: Some Additional Facts and Clarifications”

**DOI:** 10.2196/jmir.4786

**Published:** 2015-09-18

**Authors:** Marlon Perera, Matthew Roberts, Nathan Lawrentschuk, Damien Bolton

**Affiliations:** ^1^Department of SurgerySchool of MedicineUniversity of QueenslandToowoombaAustralia; ^2^Depeartment of SurgeryAustin HospitalUniversity of MelbourneMelbourneAustralia; ^3^Centre for Clinical ResearchThe University of QueenslandBrisbaneAustralia; ^4^Austin HospitalLudwig Institute for Cancer ResearchMelbourneAustralia; ^5^Division of Cancer SurgeryPeter MacCallum Cancer CentreMelbourneAustralia

We read with great interest the recent correspondence from Topf et al [1] regarding our recent publication “Globalization of Continuing Professional Development by Journal Clubs via Microblogging: A Systematic Review” [2]. We thank the authors for their interest, opinions, and contribution to the ongoing work evaluating the utility of Twitter-based Journal Clubs in the context of continuing professional development.

Topf et al note the limitation associated with the dynamic nature of the "*impressions" *data as a reported outcome measure and provide a well-explained example of how this metric is dynamic. Further, they correctly note that "spam" accounts associated with the journal clubs (JC) artificially increase the total impressions for a Twitter journal club. Despite this, when used appropriately, we believe there is some value to impressions as a performance metric given the paucity of comparative outcome measures in the early Twitter-based journal club era. This education tool is unique and traditional analysis methods typically used in systematic reviews and meta-analytical studies are clearly not suitable. The  "*impression:tweet ratio"*  reported in the initial manuscript was a metric applied only to the Twitter user account to assess the following and “impression” of the journal club, thus reducing the influence of such "spam" accounts to a degree. We believe this modified calculation is a useful quantifiable measure of publicity and potential viewership of the discussion. However, for trend analysis, such as that performed for the top five performing journal clubs, the identification and exclusion of such accounts (eg, @brodalumab) was performed as they were not only statistical outliers but also known spam accounts. This helped us to provide highly accurate data in this analysis.

The dynamic nature of Twitter-based JC was pertinently raised by the authors, as evidenced by the commencement of recent JCs. We support the notion of a "living" systematic review, not currently possible given the publication using traditional peer-reviewed methods and associated delays. The suggested method of “Storifying” the chat is an appealing method for consolidation and formalization of the conversation for later review. The value of these conversations for scholarly activity is gaining momentum, with some institutions promoting Altmetric scores for affiliated publications. Furthermore, Symplur in this context as a real-time aggregate database is an invaluable tool in appreciating the changes in journal club discussions. We anticipate that with further time and refinement, more sophisticated methods for measuring journal club performance will be devised. The ongoing success of current and future journal clubs will be determined by appropriate identification and recommendation from experienced participants with advice for successes and pitfalls from established JCs.

**Figure 1:**
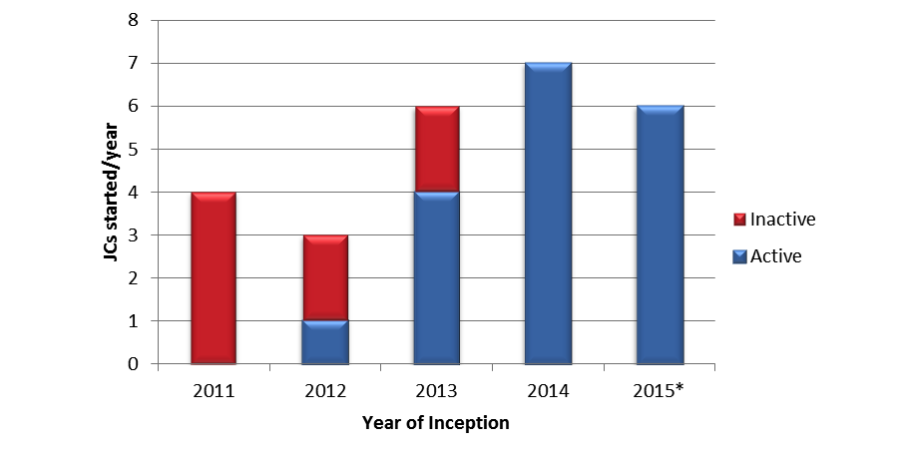
Establishment of JCs per year, comparing active JCs (blue) with inactive JCs (red). 2015 included JCs started prior to May 2015.

Given the current opportunity to present updated data six-months following the previous review [2], 6 more Twitter journal clubs have been established and none have become inactive (see Figure 1). These new journal clubs represent diverse groups within the medical field including rheumatology (#rheumJC), radiology (#medradJClub) and neuro-crictical care (#NCSTJC). Additionally, several recent publications regarding the use of social media for medical education, specifically journal clubs, have become apparent. Of these, several represent publication of summaries of a recent Twitter-based journal club discussion [3-5] or narrative reviews on the evolution of Twitter-based journal clubs [6]. Further recent publications have assessed the uptake of Twitter-based journal clubs by respective societies [7,8].
